# Positive Impact of a New Compressive Garment in Patients with Genital Lymphedema: OLYMPY Study

**DOI:** 10.1089/lrb.2023.0055

**Published:** 2024-04-17

**Authors:** Sandrine Mestre, Stéphane Vignes, Julie Malloizel-Delaunay, Sarah Abba, Stéphanie Villet, Astrid Picolet, Eric Vicaut, Isabelle Quéré

**Affiliations:** ^1^ Department of Vascular Medicine, Montpellier University, Montpellier, France.; ^2^ UA11 INSERM–UM Institut Desbrest d'Épidémiologie et de Santé Publique (IDESP), Montpellier, France.; ^3^ Department of Lymphology, Referral Center for Rare Vascular Diseases, Cognacq-Jay Hospital, Paris, France.; ^4^ Department of Vascular Medicine, Rangueil Hospital, Toulouse, France.; ^5^ Thuasne, Saint-Etienne, France.; ^6^ Clinical Research Unit, Saint-Louis, Lariboisière, Fernand-Widal Hospital, Paris, France.

**Keywords:** lymphedema, genital lymphedema, compression garment, quality of life

## Abstract

**Purpose:**

Genital lymphedema is a chronic debilitating condition associated with highly impaired health-related quality of life (QoL). This prospective multicenter study evaluated the use of a new compressive garment in patients with secondary and primary genital lymphedema.

**Methods:**

Thirty-two patients prospectively enrolled were advised to wear the compressive garment for 12 weeks (day and night). The primary endpoint was change in patient-reported QoL at 12 weeks via the patient global impression of change (PGI-C) instrument. Secondary outcomes included change in other QoL measures at 12 weeks (visual analog scale, Lymphedema Quality of Life Inventory [LyQLI], and EQ-5D questionnaires), lymphedema severity (genital lymphedema score [GLS]), and physician assessment (Clinical Global Impression–Improvement [CGI-I]). Safety and tolerability were also assessed.

**Results:**

After 12 weeks, improvement was reported in 78.6% of patients (PGI-C). Physician assessment (CGI-I) indicated clinical improvement in 82.8% of patients. Patient assessment of lymphedema symptoms showed a significant decrease in discomfort (*p* = 0.02) and swelling (*p* = 0.01). Significant declines in the mean global GLS (*p* < 0.0001), and in the proportion of patients reporting heaviness, tightness, swelling, or urinary dysfunction (*p* < 0.05 for all), were also observed. LyQLI scores decreased (indicating improved QoL) in each of the physical, psychosocial (*p* = 0.05), and practical domains. The compressive garment was well tolerated with high compliance, and adverse events (due to swelling or discomfort) led to permanent discontinuation in only three patients.

**Conclusion:**

The use of a new genital compression garment over 12 weeks improves the QoL and clinical measures in patients with genital lymphedema (ClinicalTrials.gov ID: NCT04602559; Registration: October 20, 2020).

## Introduction

Genital lymphedema is a progressive, chronic, and highly debilitating condition.^1^ Such lymphedema can be broadly considered as primary due to constitutional developmental abnormalities of the lymphatic system, or secondary after cancer treatment in high- and medium-industrialized countries, and inflammatory/infectious causes such as filariasis in tropical countries.^2,3^ Primary lymphedema can have a genetic component, usually present in younger patients and typically involves the lower extremities.^4^ Gynecologic (cervical, endometrial, ovarian, vulval/vaginal) cancers are most frequent causes of secondary genital lymphedema in females, whereas prostate and penile cancers are important causes in males; common causes in both sexes include bladder cancer and lower limb melanoma.^5–14^ In these cancers, surgical lymph node dissection, external radiation therapy, and brachytherapy are the greatest risk factors for lymphedema development.^6–11,13–15^

Clinical characteristics of genital lymphedema are heterogeneous; most patients have swelling in affected genital (penis, scrotum in male, labia in female), pubic, or abdominal sites, with lower limb involvement in most of the patients. Skin changes, for example, papillomatosis and lymphangiectasia with or without lymphorrhea are frequent, while cellulitis is an important, often recurring complication.^16,17^ In addition to the disfiguring and psychosocial impact, swelling interferes with routine physical activities; urinary and sexual dysfunction is significant and may have a substantial impact on health-related quality of life (QoL).^18–20^

Management of pelvic and genital lymphedema is also very challenging. Decongestive lymphedema therapy (DLT) is the mainstay of lymphedema management,^21^ and while some core components such as multilayer bandages, manual lymphatic drainage (MLD), skin care, exercise, therapeutic education follow well-established principles. The benefit of conventional compression therapy via bandaging or pantyhose, a critical element to DLT,^21^ is hindered by anatomical constraints in the affected pelvic area. Specific guidance for use is limited,^22^ in part as efficacy data of compression techniques in the genital lymphedema setting are lacking, and there remains a need for newer systems tailored toward the pelvic/genital region. With this aim, a new compressive garment (MOBIDERM Intimate Shorts^®^) was developed. The purpose of the present article was to report observations from a cohort study, which examined QoL and clinical outcome measures associated with the use of this device in patients with genital lymphedema.

## Methods

### Study design and patient population

This was a multicenter, prospective, open-label single-armed study (NCT04602559) conducted within three specialist centers for vascular medicine (CHU Montpellier, CHU Toulouse, and Cognacq-Jay Hospital, Paris, France) between December 2020 and July 2021. Eligible patients were individuals aged ≥15 years with secondary genital lymphedema or primary (stage II or III as defined by the International Society of Lymphology criteria^21^) presenting discomfort related to lymphedema, able to provide informed consent and participate over the 12-week period of the study follow-up. Exclusion criteria included those patients with recent genital surgical interventions in the past 3 months or intensive lymphedema reduction therapy within 4 weeks, pregnancy or planned pregnancy, or recognized contraindications for compression therapy, for example, lower limb arteriopathy.

Upon enrolment (day 0), participants were instructed to use the new compressive garment and to continue with conventional lower limb lymphedema management if initiated before the start of the study. Subjects were followed up for 12 weeks with clinical assessment at weeks 4 and 12. The study was conducted in accordance with the Declaration of Helsinki and the International Conference on Harmonization Good Clinical Practice Guidelines. The study protocol, consent form, and supporting documentation were approved by an independent ethical committee (CPP Sud-Est V) and authorized by the French Health Authority (ANSM). Written informed consent was obtained from each patient before enrolment.

### Compressive garment description

MOBIDERM Intimate Shorts (Thuasne, Saint-Etienne, France) is an innovative compression shorts, designed as an underwear developed for the management of genital edema. The maximal compression is targeted to the pelvic and genital areas. The garment consists of washable compressive shorts with different compression and support properties, available in dedicated male and female versions (each in different sizes). Within this lies a removable MOBIDERM foam pad composed of noncontiguous foam cubes. Patients were provided with a minimum of four compressive garments and instructed to wear the compressive shorts both at daytime and at night; while the foam pads are removable, patients were advised to use these for as long as possible throughout the day and overnight over the 12-week period.

### Outcome measures

Baseline characteristics were assessed at inclusion. The patient global impression of change (PGI-C) questionnaire was used to evaluate the changes observed in terms of limitation of activities, symptoms, emotions, and overall QoL after wearing the garment for 12 weeks. Patients maintained a daily diary to record compliance with shorts and pad wear instructions (in terms of days and hours per day). There is no precise definition of compliance for pressure garment wear in the literature. We defined compliance as good when use was ≥5 days each week with a minimum of 6 hours during daytime and 6 hours overnight. Safety and tolerance data were collected via the patient's diary and recorded by the investigating physicians during follow-up visits. At these visits, all data were recorded by the physicians in electronic case report forms.

The primary outcome was the patient's global impression of change in QoL at 12 weeks as assessed by the PGI-C questionnaire, frequently used in assessment of perceived impact of management on symptom control in chronic conditions.^23,24^ The PGI-C measures change in a patient's overall status on a 7-point verbal rating scale, ranging from “no change or condition has gotten worse” (1) to “a great deal better and a considerable improvement that has made all the difference” (7). As well as indicating any improvement, patients were asked to rate any change in their status on a −5 to +5 scale.

Secondary outcomes included patient and physician assessments at baseline and at weeks 4 and 12. Patient assessment of discomfort and swelling, tightness, and feeling of heaviness were measured using a visual analog scale (VAS) rated from 0 to 10 where 0 corresponds to no discomfort/no problem and 10 to maximum discomfort/problem. Change in QoL measures was assessed using different QoL instruments. The Lymphedema Quality of Life Inventory (LyQLI) was chosen as a lymphedema-specific QoL instrument.^25–28^ This questionnaire comprises a series of 41 questions spanning 3 domains (physical, psychosocial, and practical) rated 0 to 3 on a 4-point Likert scale (with higher scores reflecting greater negative impact on QoL); 4 additional questions examine overall impact on mood and QoL, where higher scores reflect better emotional mood and QoL.^25–28^

EQ-5D-5L was chosen as a generic QoL instrument that can provide a simple score, relatable to a broad range of medical conditions. EQ-5D-5L comprises five dimensions: mobility, self-care, usual activities, pain/discomfort, and anxiety/depression, each with five levels (ranging from no problems to extreme problems) with responses generating the patient's health state that can be transformed into an index score ranging between 0 (death) and 1 (perfect health).^29^ Patient scores were also assessed using the allied EQ-VAS.^30^ Impact of lymphedema on pain and on sexual relations was each rated via specific questionnaires and reported on VAS. Sleep disturbance was assessed using the Jenkins Sleep Scale questionnaire.^31^

Physician assessment of lymphedema severity was using the genital lymphedema score (GLS) questionnaire.^32^ This examines six items: feeling of heaviness, tightness, swelling, and presence of urinary disorders, lymphatic papillomatosis, and genital lymphorrhea, and correlates well with clinical severity staging systems.^32^ Evolution of scrotal perimetry in males was measured using methods described by Whitaker.^33,34^ Skin condition was evaluated by grading skin softness (ranging from indurated to very soft) in the genital region at inclusion and at subsequent visits. Physician assessment of clinical improvement was evaluated via the Clinical Global Impression–Improvement scale (CGI-I).^35^

### Data analysis

Descriptive analyses were performed for all study variables. Continuous variables are presented as mean values ± standard deviation or median and interquartile range (IQR), that is, first quartile to third quartile (Q1–Q3). Categorical variables are presented as frequency counts and percentages. Analyses were performed for intention-to-treat (ITT) and the per-protocol (PP) populations. The ITT population included all patients who wore the investigational garment at least once and who attended for at least one post-baseline visit. The PP population included all patients of the safety population without major deviations. Safety analyses were conducted in the entire population. Poor compliance, that is, wearing the garment for <4 days a week (or for <50% of the day) was considered a major protocol deviation.

As the literature is very poor on the subject and no study reference is available, no formal sample size calculations were performed. Changes in outcome measurements between day 0 and weeks 4 and 12 were assessed using the analysis of variance (ANOVA) using a mixed model for normally distributed data, and the Friedman test for nonparametric distributions. The resultant *p*-values were not adjusted for multiplicity and are reported primarily for illustrative purposes. All data analyses were performed using SAS version 9.4 (SAS Institute, Inc., NC).

## Results

### Study population

A total of 32 patients were included: 19 with secondary lymphedema (59.4%) and 13 with primary lymphedema (40.6%). The patients mean age was 60.2 ± 15.6 years (range 18–83 years) with slightly more males (*n* = 17, 53.1%); the mean body mass index was 26.7 ± 6.7 kg/m^2^ (range 16.5–48.4 kg/m^2^) and 59.3% were overweight (31.2% obese) (Table 1). Lymphedema duration was 5.4 ± 5.4 years in patients with secondary lymphedema and 12.7 ± 11.9 years in those with primary lymphedema. Most patients had stage IIB (53.1%) or stage III (43.7%) lymphedema, with associated lower limb lymphedema in 93.7% (bilateral in 68.7%). Previous treatment included MLD in 62.5% of patients, use of a compressive garment in 52.5%, of which 40% used a tailored garment, and pad use by 14 patients (43.8%). Seven patients (21.8%) had prior surgical management of genital lymphedema.

**Table 1.** Baseline Demographics and Clinical Characteristics of the 32 Patients

Characteristic	At inclusion (*n* = 32)
Age, years, mean ± SD [range]	60.2 ± 15.6 [18–83]
Sex, male, *n* (%)	17 (53.1)
BMI, kg/m^2^, mean ± SD [range]	26.7 ± 6.7 [16.5–48.4]
Underweight (<18.5), *n* (%)	3 (9.4)
Normal weight (18.5–24.9), *n* (%)	10 (31.3)
Overweight (25–29.9), *n* (%)	9 (28.1)
Obese (>30), *n* (%)	10 (31.2)
Lymphedema stage, *n* (%)	
Stage IIA	1 (3.1)
Stage IIB	17 (53.1)
Stage III	14 (43.7)
Associated lower limb lymphedema, bilateral, *n* (%)	30 (93.7), 22 (68.7)
Previous cellulitis, *n* (%)	16 (50.0)
Secondary lymphedema	19 (59.4)
Time since secondary lymphedema diagnosis, years ± SD	5.4 ± 5.4
Age at secondary lymphedema diagnosis, years ± SD	58.4 ± 13.9
Time between cancer diagnosis and secondary genital lymphedema diagnosis, years ± SD	3.6 ± 8.3
Abdominal–pelvic cancer affected organ^a^	
Uterine/cervical/ovarian, *n* (%)	12 (66.7)
Prostate, *n* (%)	5 (27.8)
External genital, *n* (%)^b^	4 (22.2)
Bladder, *n* (%)	1 (5.6)
Other, *n* (%)^c^	2 (11.1)
Cancer treatment^d^	
Surgery, *n* (%)	16 (88.9)
External radiotherapy, *n* (%)	13 (72.2)
Brachytherapy, *n* (%)	6 (33.3)
Chemotherapy, *n* (%)	11 (61.1)
Hormonal treatment, *n* (%)	3 (16.7)
Primary lymphedema	13 (40.6)
Time since primary lymphedema diagnosis, years ± SD	12.7 ± 11.9
Age at primary lymphedema diagnosis, years ± SD	40.6 ± 21.7

^a^ Percentages calculated in those patients with abdominal–pelvic cancers (*n* = 18); patients may have more than one organ affected.

^b^ Vaginal/vulval/penile/anogenital involvement.

^c^ Anorectal/renal involvement.

^d^ Percentages calculated in those patients with abdominal–pelvic cancers (*n* = 18); patients may have received multiple therapies.

BMI, body mass index; SD, standard deviation.

All the patients with secondary genital lymphedema had prior cancer diagnosis and therapy. The mean time between cancer diagnosis and onset of genital lymphedema was 3.6 ± 8.3 years. In females, most cancers were gynecologic: cervical (*n* = 7), endometrial (*n* = 3), ovarian (*n* = 2), and vulva/vagina (*n* = 3), although some patients had more than one affected site. In men, previous cancers were prostate (*n* = 5), bladder (*n* = 1), renal (*n* = 1), or penis (*n* = 1). In most patients, surgical treatment with lymph node resection had been performed (*n* = 16, 88.9%), with radiation therapy also common, either external (*n* = 13, 72.2%) or brachytherapy (*n* = 6, 33.3%). Associated chemotherapy was reported in 61.1% of patients and hormonal therapy in 16.7% of patients.

### Patient disposition and additional therapy

Of the 32 patients included at baseline, 30 patients attended for assessment at week 4, and 29 patients at week 12 and constituted the ITT population. Nine patients were excluded from the PP population for major protocol violations (inadequate compliance with garment wear duration, that is, for <4 days a week/<50% of the time), or due to study discontinuation.

All the patients continued with existing conventional lymphedema care throughout the study. At week 12, almost two thirds of the patients (65.5%) reported regular MLD on a biweekly basis. Patients with lower limb lymphedema continued to wear compression stockings.

### Patient-reported outcomes

**FIG. 1.**
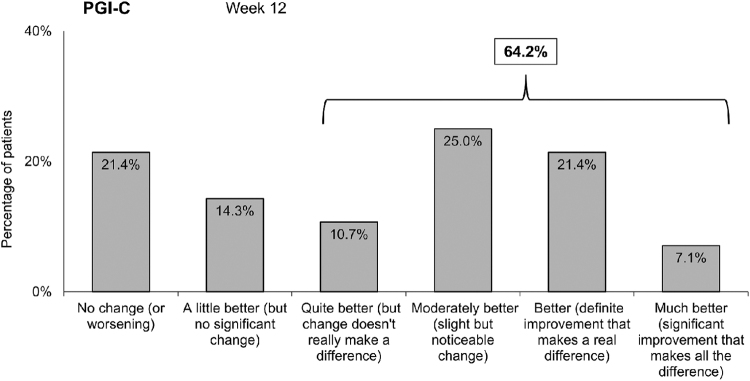
Patient assessment of improvement at 12 weeks in the ITT population. Patient assessment was performed via the PGI-C scale. Data represent the percentage of patients with improvement graded according to the PGI-C categories (week 12). ITT, intention-to-treat; PGI-C, patient global impression of change.

For the primary endpoint (PGI-C), data were available for 29 patients at week 4 and 28 patients at week 12. After 4 weeks, 76.7% of patients reported some improvement of their condition, with 16.6% indicating a strong improvement with a real relevant change. At 12 weeks, a slightly greater proportion of patients showed some improvement in PGI-C (78.6%): 14.3% reported a slight improvement, 35.7% moderate improvement (including quite better and moderately better), and 28.5% reported a strong improvement (including better and much better) (Fig. 1). The mean change was an improvement of 2.5 ± 1.6 points (median 3.0, IQR 1.5–4.0) on a −5 to +5 scale. For those patients indicating no change or worsening (21.4%), none reported a negative change on this scale that would correspond to worsening of their condition.

The primary endpoint was also assessed in the PP population (those patients without any major protocol deviations) where some improvement was reported in 91.3% at week 12; a strong improvement was reported by 34.8%, with 8.7% indicating a significant improvement that makes all the difference. The mean change was an improvement of 2.9 ± 1.4 points (median 3.0, IQR 2.0–4.0).

**FIG. 2.**
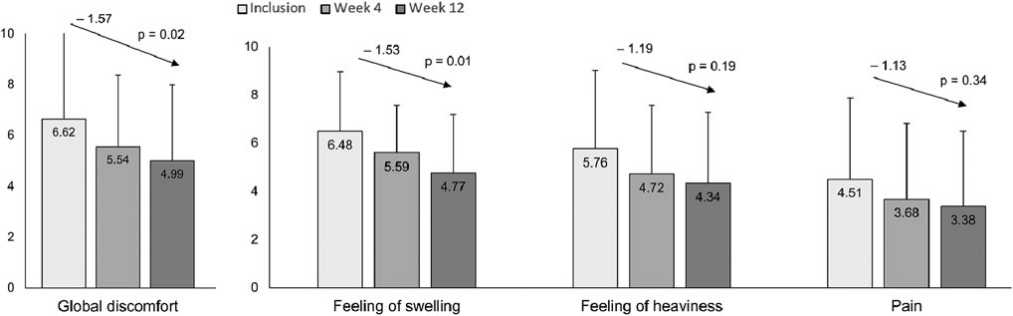
Change in patient assessment of lymphedema (VAS). Patient assessment of lymphedema symptoms was recorded on a 10-point VAS. VAS, visual analog scale.

Patient assessment of lymphedema-associated symptoms showed a consistent decrease across the study period, with mean VAS scores decreasing by over 1.5 points after 12 weeks for both global discomfort (*p* = 0.02) and swelling (*p* = 0.01) (Fig. 2). Reductions in discomfort were most apparent when patients were sitting or in a standing position. Patient VAS rating of feeling of heaviness and pain scores also declined, although these were nonsignificant (Fig. 2). Improvements in lymphedema-specific QoL and global QoL scores were observed. At 12 weeks, scores decreased (indicating improved QoL) in each of the LyQLI physical (−0.219, *p* = 0.1099), psychosocial (−0.221, *p* = 0.05), and practical domains (−0.143, *p* = 0.6842*)*, while global QoL assessed via EQ-5D VAS showed a 9.5-point increase (indicating improved QoL) from baseline (*p* = 0.18). When evaluated using the EQ-5D-5L health state questionnaire, 60.7% of patients in the ITT population reported a better QoL at 12 weeks.

Sleep quality remained relatively similar across the study period (with mean scores of 8.25 and 8.11 at baseline and week 12, respectively). There was little change on self-reported impact of lymphedema on discomfort during sexual activity (data not shown).

### Physician assessment outcomes

**FIG. 3.**
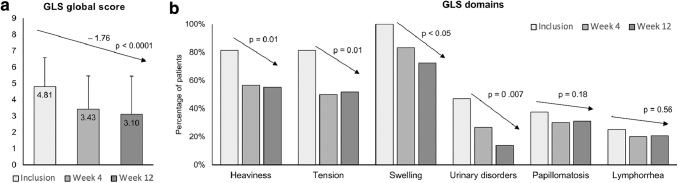
Change in GLS. **(a)** The mean global GLS at inclusion, week 4, and week 12. **(b)** The mean subscores corresponding to the six domains of the GLS scale: heaviness, tension/tightness, swelling, urinary disorders, papillomatosis, lymphorrhea at inclusion, week 4, and week 12. GLS, genital lymphedema score.

**FIG. 4.**
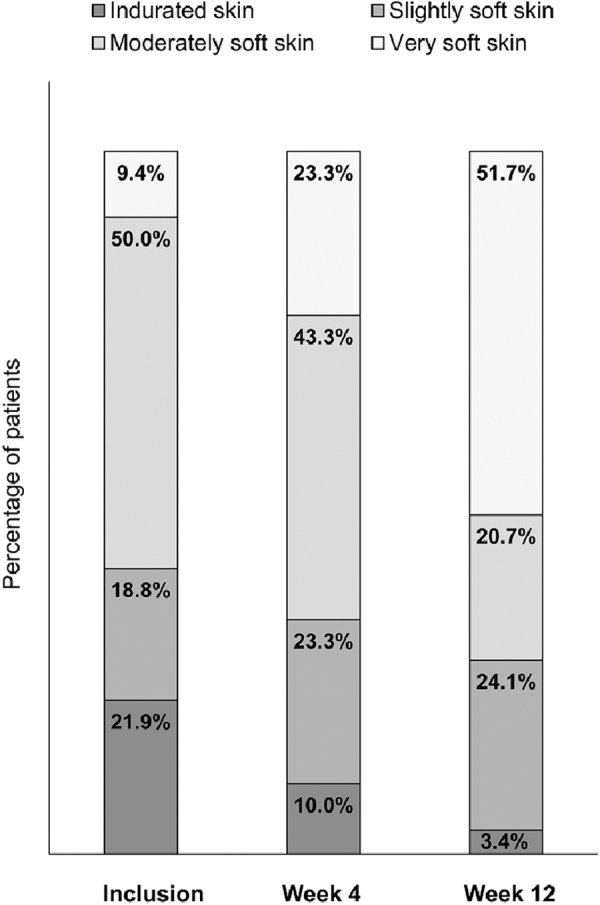
Physician assessment of skin characteristics. Skin softness was graded as indurated, moderately soft, slightly soft, or very soft at inclusion and at 4 and 12 weeks of follow-up.

A reduction in lymphedema severity was observed. The mean global GLS declined from 4.81 at baseline to 3.43 at week 4 and 3.10 at week 12, a 1.76-point reduction (*p* < 0.0001). After 12 weeks, the proportion of patients reporting heaviness, tension (or tightness), swelling, or urinary dysfunction showed substantial decline from baseline (Fig. 3). In men, scrotal perimetry indicated a reduction in middle-scrotal circumference (by 2.7 cm at week 12; *p* = 0.001) with 65% of clinicians indicating improved scrotal anatomy. Physician assessment of skin condition indicated improvement in 65.5% of patients; the proportion of patients with skin induration fell from 21.9% at inclusion to 3.4% at week 12, with a notable increase in the proportion of patients with very soft skin at this time point (Fig. 4). After 12 weeks, improvement in skin condition was observed in 65.5% of patients (*p* = 0.009).

**FIG. 5.**
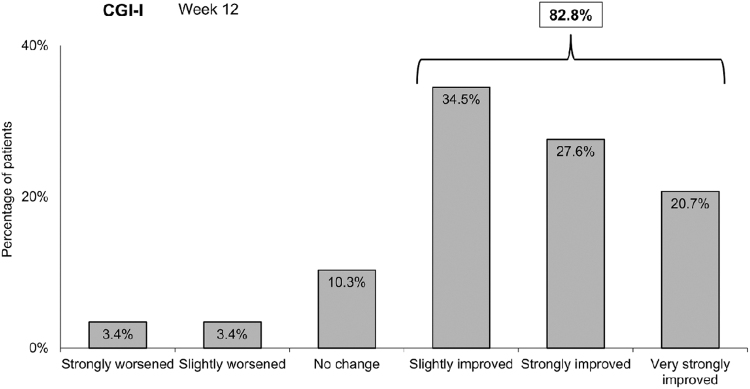
Physician assessment of improvement at 12 weeks in the ITT population. Physician assessment was performed via the CGI-I scale. Data represent the percentage of patients with improvement graded according to the CGI-I categories (week 12). CGI-I, Clinical Global Impression–Improvement.

Physician-reported CGI-I indicated their perception of clinical improvement as early as week 4; after 12 weeks, clinical improvement was observed in 82.8%, strongly improved in 27.6%, and very strongly improved in 20.7% (Fig. 5).

Patient compliance with garment wear was relatively high throughout the study, with 69% (*n* = 22) of patients wearing the compression shorts for ≥5 days each week (for at least 50% of the day) with 45% reporting overnight use for ≥5 nights each week (for at least 50% of the night). Concomitant use of the removable foam pad was also high, reported by 59% of patients for daytime use and 41% overnight. Patient satisfaction was high, with more than 80% of patients rating the compressive garment as easy to put on and take off and 86% expressing overall satisfaction. At the end of the study, most patients (96%) indicated their choice to continue with use.

### Safety and adverse events

During the study, 23 patients (71.8%) experienced at least one adverse event, including 2 serious adverse events, with a total of 30 adverse events. Neither of the serious adverse events was considered related to the study device and neither led to permanent study discontinuation. Thirteen adverse events (in 12 patients) were considered to be related to the study garment. Of these, five adverse events (38.5% of all device-related events) included skin complaints, for example, pruritus, irritation, and skin marks were reported, and five were related to lower limb or knee swelling (occurring in four patients [12.5% of all patients]). Of those adverse events considered by physicians not to be related to the garment, cellulitis was reported in 7 of 32 (21.9%) patients, most of whom (86%) had a history of cellulitis (involving genitals or lower limb).

None of which led to any discontinuations. Adverse events led to temporary discontinuation of garment wear in five patients (chiefly related to swelling of the lower limb or knee), and permanent discontinuation in three patients (due to swelling or discomfort). The first permanent discontinuation, after 5 days, was due to a localized edema in the thigh (a feature experienced with previous compressive garments) with this patient preferring to use a short stretch bandage. The second discontinuation (after 1 month) was due to reported discomfort with the largest available garment size, and the third discontinuation was due to pruritus and increased maceration, attributed to the garment's synthetic material.

## Discussion

While case series on genital lymphedema are occasionally reported,^36,37^ no previous study has evaluated the effects of compressive therapy specifically focused toward the genital region. This study is the first one that evaluated the impact of a specific tailored garment, designed to apply targeted compression to the genital and pelvic areas and to facilitate fluid treatment from the genital region. The use of the garment over 12 weeks led to improvements in a range of clinical outcomes associated with improved QoL.

Genital and pelvic lymphedema is a common and challenging condition that causes long-lasting physical, emotional, and social problems, leading to impaired QoL for affected patients and families.^5–14^ The impact of genital lymphedema is broad, including pain during walking, itching, discomfort during sleep and intercourse, lymphorrhea, and difficulty in urination. Furthermore, there remains a lack of consensus as to optimal management strategies. At present, the mainstay of management is compression therapy chiefly directed toward associated lower limb lymphedema with DLT.^19,22,38,39^

Surgical management is required for more severe/refractory cases, ranging from partial to more widespread excision of affected genital tissue (e.g., majora/minora, vulval, and scrotal/penile tissue).^36,40–42^ However, post-surgical recurrence is observed, and revision surgery may be necessary. Other treatments, such as yttrium–aluminum–garnet or CO_2_ laser, cryotherapy, or sclerotherapy, have also been used.^43^

In our study, we chose a wide range of specific measures, including patient and physician global assessments of benefit (via PGI-C and CGI-I) to capture both these important perspectives. This was influenced in part by the relative lack of standardized approaches to evaluating outcomes in genital lymphedema (including recognized difficulties in objective measures such as excess volume and volume reduction in the genital region). For our lymphedema-specific QoL measures, we chose to use the LyQLI rather than other instruments such as the Lymphedema Quality of Life Questionnaire (LYMQOL) or leg-specific versions,^30,44^ as the LyQLI can be applied to most lymphedema settings (upper and lower limb) and also regional lymphedema such as genital lymphedema.^25–28^ In addition, we evaluated generic QoL via the established EQ-5D health state questionnaire and EQ-5D VAS with benefits observed with all these QoL measures.

The garment was well tolerated, and while cellulitis was observed in a number of patients, a history of cellulitis was reported for 50% of the study population. Such cellulitis is a well-recognized complication of lymphedema in the lower limb^17^ and also reported in other studies with genital involvement.^45^

Our study has several limitations. Our study cohort was relatively small, with a heterogeneous and nonrandomized population. Our results are primarily descriptive, and the sample size limits any robust evaluation of statistical significance (although we have considered it of value to report on some for illustrative purposes). Other therapy, including continued DLT with MLD, may also have interfered in our findings (although these were already in place before study initiation; and where patients only continued existing DLT). While benefit may have included changes in lymphedema of the lower limbs, evaluation of this *per se* was not part of our study aims and evaluations. Finally, the paucity of studies evaluating garments in the management of genital lymphedema makes direct comparison with previous studies difficult.

It should also be mentioned that the present study was conducted at a period (winter 2020) in which social restrictions were again in place due to resurgence of the ongoing COVID-19 pandemic. Despite this, recruitment of 32 patients across the 3 specialized centers over a 3-month period may indicate that patients with genital lymphedema have a high level of interest in novel approaches to lymphedema management.

## Conclusion

Our study is the first study on genital lymphedema with a specific compression garment. Allied with the high levels of patient satisfaction, compliance, and ease of use, our overall findings indicate that the garment has potential benefits in routine use, with a favorable benefit–risk balance. The results suggest that the use of MOBIDERM Intimate Shorts provides clinical benefits to patients with genital edema and improvement in lymphedema severity and improved QoL. Further study is required to confirm our results.

## Ethical Approval

Applicable for both human and/or animal studies. Ethical committees, Internal Review Boards, and guidelines followed must be named. When applicable, additional headings with statements on consent to participate and consent to publish are also required.

All procedures performed in studies involving human participants were in accordance with the ethical standards of the institutional and/or national research committee and with the 1964 Declaration of Helsinki and its later amendments or comparable ethical standards. The study was approved by the ethical Committee of OUEST IV–NANTES (France).

## Consent to Participate

Written informed consent was obtained from all individual participants included in the study and from the parents when the patient was a teenager without objection.

## Availability of Data and Materials

The results of the analyses can be consulted on request from THUASNE, which stores the data on a secure computer server.
